# Healthcare in prison: Does it matter how it is provided?

**DOI:** 10.23889/ijpds.v8i2.2212

**Published:** 2023-09-14

**Authors:** Janet Bowstead

**Affiliations:** 1 Royal Holloway, University of London, London, United Kingdom

## Objectives

From April 2013, responsibility for healthcare services for prisoners was shifted to NHS England. Administrative survey data from English prisons covering 2000-2021 was used to identify if this change affected detainees’ experiences of healthcare quality and/or access; as well as the association with other characteristics of prison or prisoner.

## Method

Since 2000, HM Inspectorate of Prisons (HMIP) has carried out surveys of detainees as part of its inspections. This presentation will highlight the potential of these datasets by presenting substantive results of analysis on detainees’ experience of healthcare in prison. Merging datasets over time provides continuity of some variables over the whole period, with responses from up to 95,000 individuals. Variables of detainees’ assessment of the ease of access to different healthcare professionals, as well as the quality of services provided, were analysed over time and in terms of association with different types of prison and demographics of prisoner.

## Results

The HMIP data are used to inform inspections and reports, and an ESRC-funded project has now developed these administrative datasets for wider research use. With a timeframe of over 20 years, the data can be analysed on a range of policy-relevant prison issues, such as safety, preparation for release, support within prison, treatment of prisoners, and access to information, legal rights, education, exercise and healthcare. These can be associated with demographic characteristics of detainees, and functional types of prison; as well as the analysis presented here of trends over time and whether these can be aligned to distinct policy or practice changes. The policy change in healthcare provision in April 2013 is contextualised within trends of greater health needs of prisoners and differentials between prisoners.

## Conclusion

HMIP detainee survey data are now archived with the UK Data Service for research use. Cross-sectional analysis on a range of demographic factors shows differential healthcare needs and experiences, and analysis over time indicates both trends and the impact, or not, of policy changes on detainees’ experiences."

